# Epidemiology of Suicide in Older Adults

**DOI:** 10.1093/geroni/igaa057.2130

**Published:** 2020-12-16

**Authors:** Jane Pearson

**Affiliations:** National Institute of Mental Health, ROCKVILLE, Maryland, United States

## Abstract

This individual symposium abstract will focus on the epidemiology of suicide in older adults, with particular focus on risk factors, changing demographics, and population shifts with the baby-boomers aging. Epidemiologically, older men aged 75 and older have a suicide rate of 39.7 deaths per 100,000 in 2017, compared to the general population of 14.0 deaths per 100,000. Risk factors for suicide in older adults include functional disability, multiple chronic physical conditions, and social isolation. In addition, older adults often face stressors such as relationship issues, life crises (loss of spouse), and social factors (employment and financial challenges, housing stress, and legal issues). Limited mobility, physical and mental health conditions, and lack of social support can affect healthcare access and utilization. Many older adults do not routinely seek behavioral health treatment, with reported under-detection of mental health conditions such as depression, substance use disorders, and suicidal ideation.

